# Frequency and function of KIR^+^ CD8^+^ T cells in HTLV-1 infection

**DOI:** 10.1186/1742-4690-11-S1-P79

**Published:** 2014-01-07

**Authors:** Katie Twigger, Aileen Rowan, Nafisa-Katrin Seich al Basatena, Aidan MacNamara, Christelle Retiere, Keith Gould, Graham P Taylor, Becca Asquith, Charles RM Bangham

**Affiliations:** 1 Section of Immunology, Imperial College London, UK; 2 Department of Genito-Urinary Medicine and Communicable Diseases, Imperial College London, UK; 3 Immunovirologie et polymorphisme génétique, Etablissement Français du Sang, Université de Nantes, Nantes, France

## 

An efficient antiviral cytotoxic T lymphocyte (CTL) response to HTLV-1 infection maintains a low proviral load (PVL), reducing the risk of HAM/TSP. Host genotype, particularly of HLA class I, is a major determinant of CTL efficiency, and the influence of specific HLA class I alleles on HTLV-1 immunity is well documented. We recently showed that killer immunoglobulin-like receptor (KIR) genotype also influences CTL efficiency, by affecting HLA class I-mediated HTLV-1 immunity. Possession of the KIR2DL2 gene enhanced the effect of known protective or detrimental HLA class I alleles on PVL and HAM/TSP risk. This study aims to profile the frequency and function of CD8^+^ T cells expressing KIR2DL2 and other KIRs in HTLV-1 infection. Analysing total KIR expression showed the presence of KIR^+^CD8^+^ T cells, NK cells and CD4^+^ T cells in PBMCs from uninfected and HTLV-1^+^ donors. A subset of HTLV-1^+^ donors had high frequencies of total KIR^+^CD8^+^ T cells. Analysing individual KIRs revealed the presence of KIR2DL2^+^CD8^+^ T cells in HTLV-1^+^ donors. Preliminary data from PBMCs stimulated with Tax peptides indicates that KIR2DL2^+^CD8^+^ T cells constitute a very small proportion of the IFNγ-producing Tax-specific CD8^+^ T cell population. HTLV-1^+^ asymptomatic carriers had higher frequencies of IFNγ-producing Tax-specific KIR2DL2^+^CD8^+^ T cells than donors with HAM/TSP did. Further work is underway to characterise the function of Tax-specific CD8^+^ T cells by staining with anti-CD107a, anti-IFNγ and the HLA-A2/Tax_11-19_ pentamer, and to compare the frequency and function of KIR2DL2^+^CD8^+^ T cells with those expressing other 2-domain KIRs.

